# High Mobility Group AT-hook 2: A Biomarker Associated with Resistance to Enzalutamide in Prostate Cancer Cells

**DOI:** 10.3390/cancers16152631

**Published:** 2024-07-24

**Authors:** Yusuf Mansur Liadi, Taaliah Campbell, Bor-Jang Hwang, Bethtrice Elliott, Valerie Odero-Marah

**Affiliations:** 1 Center for Urban Health Disparities Research and Innovation, Department of Biology, Morgan State University, Baltimore, MD 21251, USA; yulia1@morgan.edu (Y.M.L.); bor-jang.hwang@morgan.edu (B.-J.H.); bethtrice.elliott@morgan.edu (B.E.); 2 Department of Biology, Umaru Musa Yar’adua University, Katsina 820102, Nigeria; 3 Center for Cancer Research and Therapeutic Development, Department of Biological Sciences, Clark Atlanta University, Atlanta, GA 30314, USA; taaliahqcampbell@gmail.com

**Keywords:** metastatic prostate cancer, enzalutamide-resistant, high mobility group AT-hook 2 (HMGA2), epithelial to mesenchymal transition (EMT), androgen receptor (AR), Snail, alisertib, enzalutamide

## Abstract

**Simple Summary:**

Metastatic prostate cancer (mPCa) is a leading cause of death primarily due to its resistance to treatments like enzalutamide. This study investigates how the protein HMGA2 contributes to this resistance. We found that cancer cells with high levels of HMGA2 became less sensitive to enzalutamide but not to another drug, alisertib. Interestingly, although these cells resisted enzalutamide, they did not show changes in a key prostate cancer protein called AR. Furthermore, a knockdown of HMGA2 sensitized the cells to both drugs. Clinically, 3% of patients showed changes in HMGA2, which were more common in cancers that had metastasized to bones, lymph nodes, and the liver. Our research suggests that HMGA2 could be used as a marker to predict enzalutamide resistance in mPCa cells. Additionally, alisertib may be an effective treatment for patients with high HMGA2 levels, offering a new potential therapeutic strategy for managing metastatic prostate cancer.

**Abstract:**

Metastatic prostate cancer (mPCa) is a leading cause of mortality, partly due to its resistance to anti-androgens like enzalutamide. Snail can promote this resistance by increasing full-length AR and AR-V7. High Mobility Group AT-hook 2 (HMGA2), a DNA-binding protein upstream of Snail, is crucial in proliferation and epithelial–mesenchymal transition (EMT). This study examines HMGA2’s role in enzalutamide resistance. LNCaP and 22Rv1 cells overexpressing wild-type HMGA2, but not truncated HMGA2, showed EMT. Both variants led to a decreased sensitivity to enzalutamide but not alisertib compared to Neo control cells. The overexpression of HMGA2 did not alter AR expression. Enzalutamide-resistant C4-2B cells (C4-2B MDVR) had higher HMGA2 and AR/AR variant expression than enzalutamide-sensitive C4-2B cells but remained sensitive to alisertib. The HMGA2 knockdown in C4-2B MDVR cells increased sensitivity to both enzalutamide and alisertib without changing AR expression. A clinical analysis via cBioPortal revealed HMGA2 alterations in 3% and AR alterations in 59% of patients. The HMGA2 changes were linked to treatments like enzalutamide, abiraterone, or alisertib, with amplifications more prevalent in bone, lymph node, and liver metastases. Conclusively, HMGA2 is a potential biomarker for enzalutamide resistance in mPCa, independent of Snail and AR signaling, and alisertib may be an effective treatment for mPCa that expresses HMGA2.

## 1. Introduction

Prostate cancer (PCa) is the most common non-cutaneous cancer in men globally, with an estimated 299,010 new cases expected to result in 35,250 deaths in the United States in 2024 (ACS, 2024). Despite the effectiveness of radiation therapy and surgery in treating localized PCa, metastatic disease (mPCa) recurrence occurs in 30% of patients, leading to a poor prognosis and a five-year survival rate of only 29% [1]. Most PCa-related deaths result from metastatic disease [2], commonly affecting the liver, lungs, and bones after lymph node invasion near the primary tumor [3]. The dysregulation of androgen receptor (AR) functioning is associated with PCa, and androgen deprivation therapies have been effective in blocking the action of AR, thus impeding disease progression [4]. Enzalutamide, a new-generation inhibitor, disrupts AR signaling and has demonstrated clinical efficacy in increasing overall survival and delaying disease progression in castration-resistant PCa (CRPC) [5]. Despite its initial success, resistance to enzalutamide inevitably develops [6]. Advanced PCa is usually treated with ADT, which encompasses initial treatment by suppressing testosterone levels via surgical or medical castration [7]. However, the inevitable progression of the disease to CRPC and later mCRPC informs the use of androgen-blocking therapies such as enzalutamide [8]. The AR-signaling pathway is targeted by enzalutamide by preventing androgen binding to AR, inhibiting translocation of AR to the nucleus, and preventing AR binding to the target gene [9]. Snail transcription factor has been reported to mediate enzalutamide resistance through an increase in full-length AR and AR-V7 expression and nuclear localization in PCa [10].

The pathogenesis of many malignancies, including PCa, involves an uncontrolled cell cycle driven by mutations and overexpression of various proteins. Aurora kinase A (AURKA) plays a crucial role in mitotic control and acts as an oncogene in various cancer types, including PCa [11]. AURKA is overexpressed in PCa, including CRPC and neuroendocrine prostate cancer (NEPC), demonstrating its crucial role in the progression of PCa [12]. Alisertib, an Aurora kinase A inhibitor, disrupts mitosis and spindle formation, leading to cell death [13,14]. Researchers found that in a phase II trial evaluating the effectiveness of alisertib in patients with CRPC and NEPC, alisertib led to a notable reduction in disease progression in 30% of the patients [15].

High mobility group AT-hook 2 (HMGA2), a non-histone chromatin protein, significantly contributes to PCa progression by inducing epithelial–mesenchymal transition (EMT) and promoting migration and proliferation [16]. The HMGA2 protein is overexpressed in various cancers, such as pancreatic cancer [17] and lung cancer [18], and is associated with a poor prognosis [18]. HMGA2 has also been associated with EMT and metastasis in renal carcinoma [19], nasopharyngeal carcinoma [20,21], tongue cancer [22], and oral squamous cell carcinoma [23]. Both the full-length or wild-type (WT) HMGA2 and truncated (TR) HMGA2 isoforms (without the 3′UTR) are overexpressed in various malignancies [24]. Previous studies in LNCaP PCa cells revealed that the overexpression of the HMGA2 wild-type induced EMT by activating the MAPK pathway, while the truncated HMGA2 promoted cell migration and proliferation [25]. This suggests distinct roles for the wild-type isoform in EMT and the truncated isoform in proliferation and migration.

Our study investigates the potential role of HMGA2 as a biomarker associated with enzalutamide resistance in PCa.

## 2. Materials and Methods

### 2.1. Cell Lines and Tissue Culture

PCa cell lines, including LNCaP, C4-2B, and 22Rv1, were obtained from the American Type Culture Collection (Manassas, VA, USA). C4–2B MDVR cell lines were generously provided by Dr. Allen Gao (University of California, Davis, CA, USA). LNCaP cells expressing HMGA2 wild-type (WT), HMGA2 truncated (TR), and LNCaP Neo were obtained through successful prior transfections using HMGA2 wild-type plasmid, HMGA2 truncated plasmid, and empty vector pcDNA 3.1 [14]. All cells were cultured in RPMI-1640 (Corning, Manassas, VA, USA), supplemented with 10% (*v*/*v*) FBS from GeminiBio (West Sacramento, CA, USA), 50 g/mL penicillin, and 100 g/mL streptomycin. All cells were maintained in a humid incubator at 37 °C with 5% CO_2_. 

### 2.2. Reagents and Antibodies

A nitrocellulose membrane (Catalog #: 10600002) was obtained from Bio-Rad (Hercules, CA, USA). An anti-mouse β-actin (Catalog #: sc-47778) was purchased from Santa Cruz Biotechnology (Santa Cruz, CA, USA). An anti-rat monoclonal Snail antibody (Catalog #: 4719S) and an anti-rabbit monoclonal AR antibody (Catalog #: 5153S) were purchased from Cell Signaling Technology (Danvers, MA, USA). An anti-rabbit monoclonal HMGA2 (Catalog #: PA5-21320) was obtained from Invitrogen Life Technologies (Waltham, MA, USA). A goat monoclonal anti-vimentin antibody (Catalog #: AF2105) was purchased from R&D Systems (Minneapolis, MN, USA). An anti-mouse monoclonal E-cadherin (Catalog #: 610182) was obtained from B.D. Biosciences. The protease inhibitor cocktail (Catalog #: 11873580001) was from Roche Molecular Biochemicals, Indianapolis. siRNA (Non-Targeting Pool, Catalog #: D-001810-10-05); HMGA2 (Catalog #: L-013495-00-0005) and DharmaFect reagent (siRNA Buffer (Catalog #: B-002000-UB-100)); and nuclease-free water (Catalog #: B-003000-WB-100) were purchased from Horizon discovery (Cambridgeshire, UK). A dextrose charcoal-stripped fetal bovine serum (DCC) was obtained from GeneTex, Inc. (Irvine, CA, USA).

### 2.3. Stable Transfection of 22Rv1 Cells with HMGA2 cDNA

Stable transfection of the 22Rv1 cells with HMGA2 WT and TR cDNA was achieved using a TurboFect transfection reagent (Catalog #: R0533) (Thermo Scientific, Waltham, MA, USA). Following the manufacturer’s instructions, cells at 90% confluency were transfected with 1.6 µg of HMGA2 WT cDNA (Catalog #: OHu25597D, GenScript, Piscataway, NJ, USA), HMGA2 TR (Catalog #: OHu102359D, GenScript) or an empty vector (Neo) in 12-well dishes. The selection of stable clones was carried out with 600 µg/mL G418, and maintenance was sustained with 300 µg/mL G418. A Western blot analysis was performed to validate the expression of HMGA2.

### 2.4. Treatments with Alisertib and Enzalutamide

A total of 2 × 10^3^ cells were plated in 96-well plates overnight. On the following day, the cells underwent 3 h serum starvation and were then exposed to various concentrations of alisertib (2.5, 5, 10, 20, and 40 µM) or enzalutamide (1, 5, 10, 20, and 30 µM) or 0.005% DMSO control diluted in 5% DCC for 72 h, before conducting proliferation assays. For the Western blot analysis, 5 × 10^5^ cells were seeded in 6-well plates overnight, serum-starved, and treated with 20 µM of the drugs for 72 h, followed by a collection of cell lysates.

### 2.5. Transfection with siRNA

The C4-2B MDVR cells were grown in 6-well plates and transfected with the HMGA2 siRNA using a DharmaFect reagent (Horizon discovery, Cambridgeshire, UK) in an antibiotic-free reduced-serum medium for 72 h, following the manufacturer’s instructions. The siRNA final concentration was 25 nM, and the knockdown efficiency was assessed using the Western blot analysis. Subsequently, for cell proliferation studies, the cells were treated with siRNA for 12 h, followed by drug treatment for 72 h.

### 2.6. Cell Viability

The cells were seeded in a 96-well tissue culture plate at a density of 2000 cells per well and allowed to adhere overnight. Following a 3 h serum deprivation, the cells were treated with the respective drugs (enzalutamide and alisertib). The viability was assessed 72 h post-drug treatment using the MTS CellTiter 96^®^ Aqueous One Solution Cell Proliferation Assay (Catalog #: G3580), following the supplier’s protocol (Promega Corp, Madison, WI, USA). The graphs were plotted as a percentage of the control group, and the IC50 was obtained as the point where 50% from the y-axis intercepts the trendline. 

### 2.7. Western Blot Analysis

A cell lysate extraction was performed using a modified RIPA buffer, as previously described [25]. Electrophoresis on 10% sodium dodecyl sulfate–polyacrylamide gel was utilized to separate 20–30 µg of cell lysate, which was then trans-blotted onto a nitrocellulose membrane. Subsequently, the membranes were treated with appropriate primary and secondary antibodies, and the results were visualized using Immobilon Forte Western HRP substrate (Burlington, MA, USA). For reprobing with a different antibody, the membranes were stripped using Pierce Biotechnology’s Restore Western blot stripping buffer (Rockford, IL, USA)

### 2.8. Immunofluorescence Assay

A total of 5 × 10^3^ cells were plated into 8-well chamber slides (Merck Millipore, Tullagreen, Germany). The cells were fixed with a mixture of methanol/ethanol (1:1 volume) and then blocked with Dako Protein block (Agilent Technologies, Santa Clara, CA, USA). Subsequently, the slides were incubated with a primary antibody at dilutions 1:50 or 1:100 in a Dako antibody diluent solution for 1 h at room temperature. Afterward, the slides were washed with 1× TBS-T (Dako, Camarillo, CA, USA) and incubated with secondary antibodies in the dark for 1 h at room temperature. The secondary antibodies used included goat anti-rabbit Oregon green 488, anti-mouse Alexa red 594 (Invitrogen, Carlsbad, CA, USA), and rabbit anti-goat Texas red (Vector Laboratories Inc, Burlingame, CA, USA). Following washing, the slides were counterstained with DAPI (Invitrogen, Carlsbad, CA, USA) and mounted using Vectashield antifade mounting medium (Vector Laboratories Inc, Burlingame, CA, USA). Fluorescence microscopy was conducted using a Leica DMi8 Stellaris 5 microscope (Leica Microsystems GmbH, Mannheim, Germany) and Leica Application Suite X software version 4.6.1.27508 was used for image acquisition.

### 2.9. Analysis of Publicly Available Datasets

To examine chemotherapeutic strategies for mPCa patients with HMGA2 amplification, we accessed data from SU2C/PCF, PNAS 2019, through cbioportal (http://cbioportal.org) accessed on 1 April 2024. Navigating to the website homepage, we selected “Query” and then chose “Prostate Cancer (SU2C/PCF, PNAS 2019)”, which included 444 samples. Within the “Select Genomics Profiles”, we opted for “Putative copy-number alterations from DNA copy” and under “Select Patient/Case Set”, we specified “All Tumors (444)”. We then entered the gene set “HMGA2” or “AR” and proceeded to “Submit Query”. Moving on to the “Plots” section, we customized the analysis further. For the horizontal axis, we selected “Clinical Attribute”, “Tissue Site” or “Abiraterone (ABI) and Enzalutamide Exposure Status”, and “Chemo Regimen Category”, while the vertical axis represented “Copy Number” and “Putative copy-number alterations”. These parameters resulted in the generation of the figures. Subsequently, we downloaded the mRNA expression values (RNA Seq RPKM, Reads Per Kilobase Million) for AR and HMGA2, as well as the Chemo Regimen Category data, from the cBioportal plot page.

### 2.10. Statistical Analysis

Data were analyzed using a paired Student’s *t*-test or ANOVA using GraphPad Prism software v. 10.2.0. All experiments were conducted at least three times independently, and the results are representative of three independent experiments.

## 3. Results

### 3.1. HMGA2 Overexpression in LNCaP and 22Rv1 Prostate Cancer Cells Is Associated with Resistance to Enzalutamide but Not Alisertib

**Figure 1:**
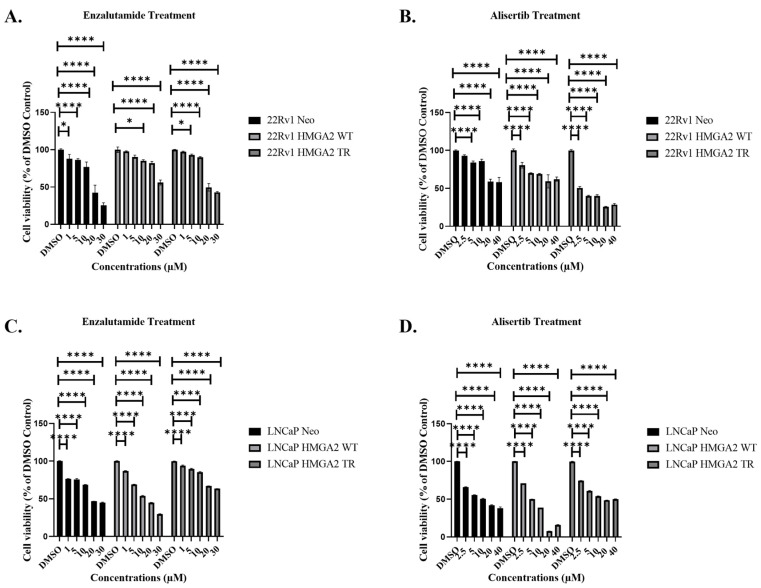
Alisertib inhibits cell viability in LNCaP and 22Rv1 prostate cancer cells overexpressing HMGA2 more potently compared to enzalutamide. Representative 22Rv1 Neo, -WT, and -TR cells were treated with (**A**) varying concentrations (1, 5, 10, 20, 30 µM) of enzalutamide or (**B**) 2.5, 5, 10, 20, or 40 µM alisertib, followed by cell viability assay 72 h later. Representative LNCaP Neo, -WT and TR cells were treated with (**C**) varying concentrations (1, 5, 10, 20, 30 µM) of enzalutamide or (**D**) 2.5, 5, 10, 20, or 40 µM alisertib, followed by cell viability assay 72 h later. Experiments were conducted at least three times independently, and the results are representative of three independent experiments. Statistical analysis was performed using GraphPad Prism, (**** *p* < 0.0001, * *p* < 0.05). Error bars show the standard deviation of the mean. The results are representative of 2 independent experiments.

To evaluate whether HMGA2 overexpression is associated with resistance to enzalutamide, we utilized LNCaP and 22Rv1 cells. The LNCaP cells were previously generated to overexpress empty vector Neo, HMGA2 WT, and HMGA2 TR [25]. In this study, the 22Rv1 cells were generated to overexpress empty Neo, HMGA2 WT, and HMGA2 TR. The Western blot analysis confirmed HMGA2, AR, and EMT marker expression in these cells (Supplemental Figure S1A). Notably, HMGA2 overexpression did not increase AR levels in the 22Rv1 cells (Supplemental Figure S1A), which shows that HMGA2 does not directly regulate AR expression in 22Rv1 cells. Representative 22Rv1 clones, WT clone 2, and TR clone 8, exhibiting increased proliferation (Supplemental Figure S1B) and partial EMT (as shown by increased Snail and vimentin for WT) (Supplemental Figure S1A) were selected for treatments. These 22Rv1 and LNCaP cell transfectants underwent treatment with various concentrations of enzalutamide (1, 5, 10, 20, and 30 µM) or alisertib (2.5, 5, 10, 20, and 40 µM), and dose-dependent growth suppression was observed (Figure 1A–D). The determined IC50 values for enzalutamide were 22Rv1 Neo = 18.5 µM, 22Rv1 HMGA2 WT > 30 µM and 22Rv1 HMGA2 TR = 19.9 µM and LNCaP Neo = 18.3 µM, LNCaP HMGA2 WT = 14 µM, LNCaP HMGA2 TR > 30 µM (Figure 1A,C). The IC50 values for alisertib were 22Rv1 Neo > 40 µM, 22Rv1 HMGA2 WT = 31.32 µM and 22Rv1 HMGA2 TR = 6.36 µM and LNCaP Neo = 10 µM, LNCaP HMGA2 WT = 5 µM, LNCaP HMGA2 TR = 15.5 µM (Figure 1B,D). Therefore, wild-type HMGA2 overexpression in 22Rv1 cells promotes resistance to enzalutamide, whereas, in LNCaP cells, the truncated HMGA2 isoforms led to greater resistance to enzalutamide compared to Neo control and wild-type overexpressing cells. This suggests some possible variations that may be due to cell line differences.

### 3.2. HMGA2 Is Associated with Resistance to Enzalutamide Independent of EMT and AR

**Figure 2:**
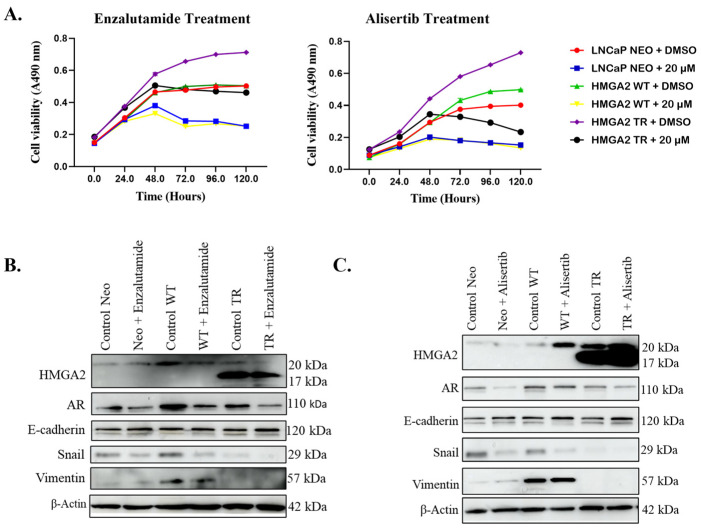
HMGA2 confers resistance to enzalutamide but not alisertib, independent of EMT and AR. LNCaP Neo, -WT, or -TR cells were treated for 0–120 h with 20 µM of (**A**) enzalutamide (**left panel**) or alisertib (**right panel**), followed by cell viability assay. Western blot analysis was performed on LNCaP cells overexpressing Neo, HMGA2-WT, or HMGA2-TR following treatment with 20 µM of (**B**) enzalutamide or (**C**) alisertib, probing for various markers associated with EMT. β-actin served as the loading control. Experiments were conducted at least three times independently, and the results are representative of three independent experiments. Statistical analysis was performed using GraphPad Prism. Error bars show the standard deviation of the mean. The results are representative of 2 independent experiments.

Subsequent time-dependent treatment with 20 µM enzalutamide for 120 h revealed effective suppression of cell viability in LNCaP Neo, LNCaP HMGA2 WT, and to a lesser extent, LNCaP HMGA2 TR cells by 72 h (Figure 2A, left panel). Moreover, treatment with 20 µM of alisertib resulted in substantial suppression of cell viability in all three cell types by 72–120 h (Figure 2A, right panel).

Having observed promising but varying antiproliferative effects of both drugs on cells expressing HMGA2, we sought to investigate the underlying mechanisms using LNCaP as a representative cell model. The Western blot analysis revealed an increase in Snail, vimentin, and AR in LNCaP HMGA2 WT cells compared to LNCaP Neo and LNCaP HMGA2 TR cells (Figure 2B). Interestingly, enzalutamide treatment led to a significant reduction in the expression of the AR protein (Figure 2B). The LNCaP HMGA2 TR cells did not display EMT (low Snail and vimentin), and enzalutamide and alisertib did not alter this (Figure 2B,C).

The alisertib treatment decreased Snail expression in LNCaP Neo and HMGA2 WT cells, with no significant change in the AR levels (Figure 2C). These findings for AR are confirmed by immunofluorescent analysis (Supplemental Figures S2–S4). Overall, the HMGA2-mediated resistance to enzalutamide in LNCaP and 22Rv1 cells is independent of EMT and AR.

### 3.3. Enzalutamide-Resistant Cells Express HMGA2 and AR Variants and Are Sensitive to Alisertib

**Figure 3:**
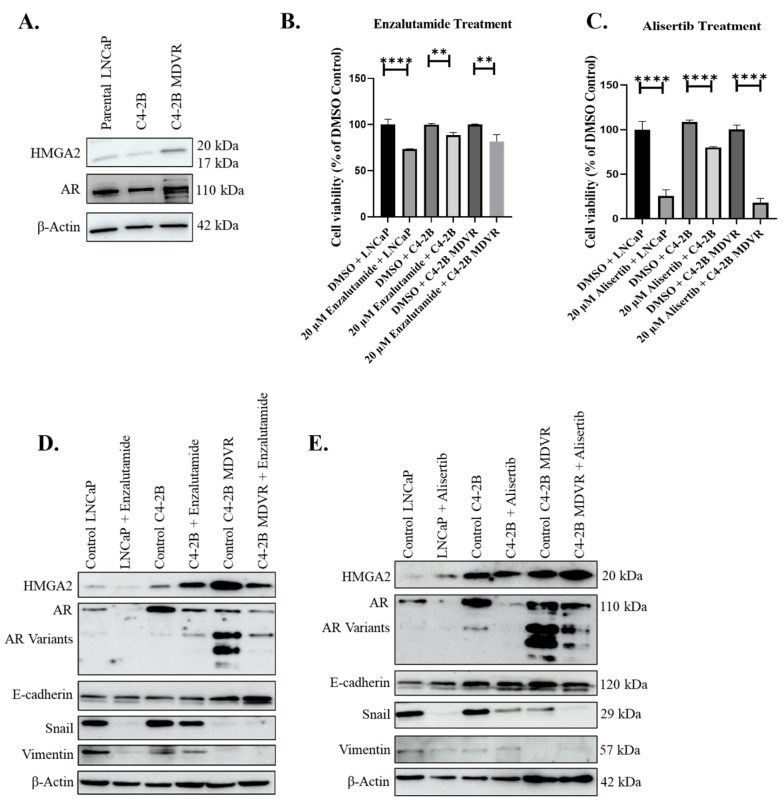
Enzalutamide-resistant C4-2B MDVR cells display higher HMGA2 and AR variants, and are sensitive to alisertib. (**A**) Western blot analysis of baseline expression of HMGA2 and AR in various PCa cell lines: LNCaP, C4-2B, and C4-2B MDVR. (**B**) Cell viability assay was performed on LNCaP, C4-2B, and C4-2B MDVR following treatment with 20 µM enzalutamide or DMSO control for 72 h. (**C**) Cell viability assay was performed on LNCaP, C4-2B, and C4-2B MDVR following treatment with 20 µM alisertib for 72 h. Western blot analysis was conducted to assess the expression levels of HMGA2, AR, E-cadherin, Snail, and Vimentin in LNCaP, C4-2B, and C4-2B MDVR cells following treatment with (**D**) 20 µM enzalutamide or (**E**) 20 µM alisertib. β-actin served as the loading control. Experiments were conducted at least three times independently, and the results are representative of three independent experiments. Statistical analysis was performed using GraphPad Prism, (**** *p* < 0.0001, ** *p* < 0.01). Error bars show the standard deviation of the mean. The results are representative of 3 independent experiments.

We sought to compare the response of PCa cells to enzalutamide and alisertib using *an* enzalutamide-resistant cell line, C4-2B MDVR. First, we sought to investigate the relationship between HMGA2 and AR expression. To assess the protein levels of HMGA2 and AR in PCa cells, we conducted Western blot analysis across various PCa cell lines, including parental LNCaP originally derived from lymph node metastasis, C4-2B, a bone metastatic subline of LNCaP, and enzalutamide-resistant C4-2B cells (C4-2B MDVR). The enzalutamide resistance cell line, C4-2B MDVR, exhibited higher protein expression levels for both HMGA2 and AR, including AR variants, as compared to the C4-2B and LNCaP cells (Figure 3A). Subsequently, we compared the drug response of these PCa cells to enzalutamide and alisertib. The concentration of 20 µM enzalutamide suppressed the growth of LNCaP and C4-2B but did not significantly suppress the proliferation of C4-2B MDVR cells compared to DMSO controls (Figure 3B). Conversely, alisertib induced a significant decline in the growth of the three cell lines, particularly in LNCaP and C4-2B MDVR cells (Figure 3C). Therefore, alisertib treatment appeared to be more potent than enzalutamide. The dose-dependent treatments of the C4-2B MDVR cells treated with varying concentrations (1, 5, 10, 20, and 30 µM) of enzalutamide, as well as varying concentrations (2.5, 5, 10, 20, and 40 µM) of alisertib for 72 h confirm that enzalutamide displays an IC50 exceeding 30 µM, whereas alisertib has an IC50 of 19.8 µM (Supplemental Figure S5).

Next, we aimed to elucidate the EMT markers in the enzalutamide-sensitive LNCaP and C4-2B and the enzalutamide-resistant C4-2B MDVR cells. We noted that the C42B MDVR cells displayed lower levels of Snail and vimentin compared to C4-2B, but higher levels of AR and AR variants (Figure 3D). Interestingly, the treatment with the 20 µM enzalutamide decreased the protein expression of Snail and vimentin in the LNCaP and C4-2B cells, while E-cadherin expression increased specifically in the C4-2B MDVR cells following enzalutamide treatment (Figure 3D). Notably, the protein expression of AR was significantly reduced in all three cell lines after treatment with 20 µM enzalutamide or alisertib, suggesting the ability of enzalutamide and alisertib to inhibit AR (Figure 3D,E). These Western blot results are confirmed using immunofluorescent analysis (Supplemental Figures S6–S8). Overall, enzalutamide-resistant C4-2B MDVR cells express higher levels of HMGA2 and AR compared to C4-2B cells and are sensitive to alisertib.

### 3.4. HMGA2 Knockdown in C4-2B MDVR Cells Promotes Sensitivity to Enzalutamide and Increases Sensitivity to Alisertib Independent of AR

**Figure 4:**
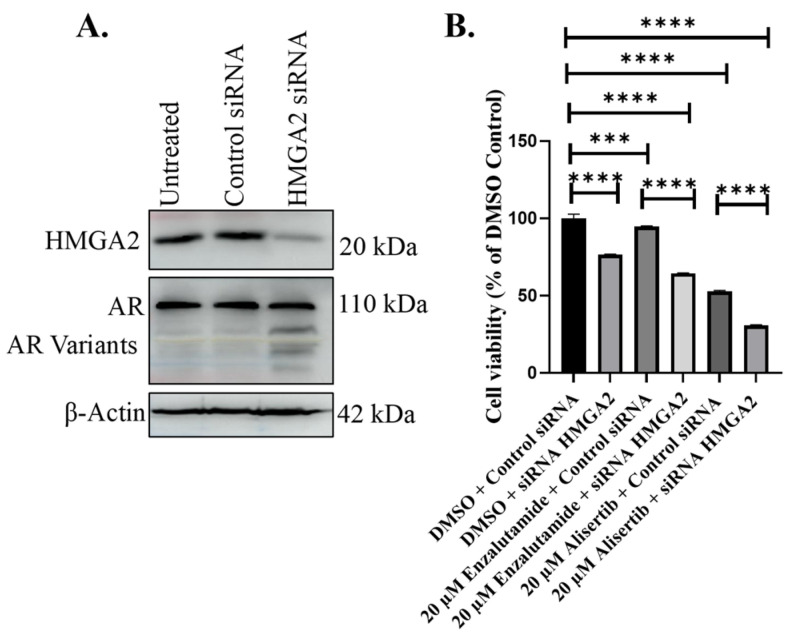
HMGA2 knockdown in C4-2B MDVR cells increases sensitivity to enzalutamide and alisertib. (**A**) HMGA2 siRNA was utilized to knock down HMGA2 in C4-2B MDVR cells, compared to control siRNA or untreated cells. Knockdown of HMGA2 in C4-2B MDVR cells was confirmed by Western blot analysis. (**B**) Viability assays were performed in C4-2B MDVR cells treated with control of HMGA2 siRNA plus DMSO control, 20 mM enzalutamide, or 20 mM alisertib. Experiments were conducted at least three times independently, and the results are representative of three independent experiments. Statistical analysis was performed using GraphPad Prism, (**** *p* < 0.0001, *** *p* < 0.001). Error bars show the standard deviation of the mean. Results are representative of 2 independent experiments.

To assess the impact of transient HMGA2 knockdown on enzalutamide response in C4-2B MDVR, we transiently transfected the cells with HMGA2 siRNA. Western blot analysis confirmed that HMGA2 siRNA decreased HMGA2 expression but not AR (Figure 4A). Subsequently, we combined the HMGA2 knockdown with 20 µM enzalutamide or alisertib, followed by the cell viability analysis. Enzalutamide and alisertib significantly reduced proliferation in C4-2B MDVR cells with HMGA2 siRNA compared to the control non-silencing group showing that HMGA2 knockdown can increase sensitivity to enzalutamide and alisertib (Figure 4B). Therefore, the HMGA2 knockdown in enzalutamide-resistant cells renders sensitivity to enzalutamide independent of AR, as well as increased sensitivity to alisertib.

### 3.5. Patients with mPCa Expressing HMGA2 and AR Alterations Received Treatment Anti-Androgen or Alisertib

**Figure 5:**
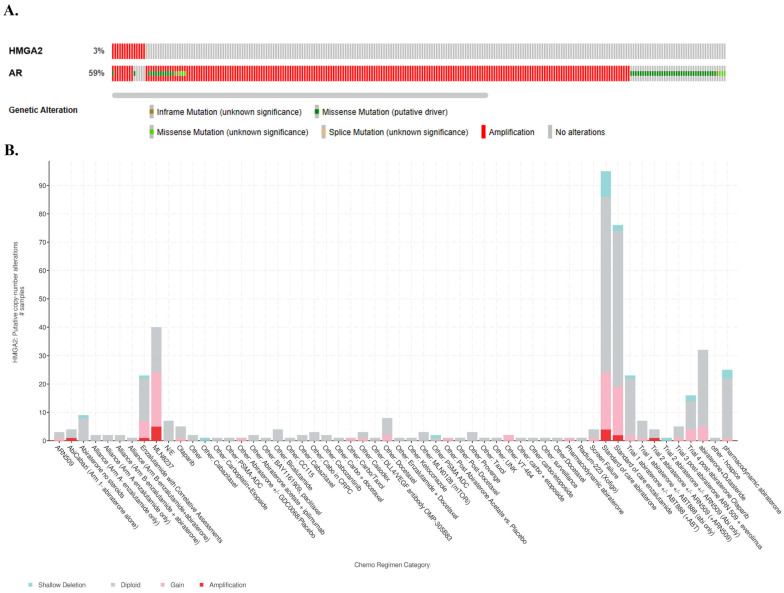
Alterations of HMGA2 and AR in metastatic PCa (mPCa) and associated treatment regimens. (**A**) OncoPrints showing genetic alterations in HMGA2 and AR. Orange represents inframe mutation, green represents missense mutation, yellow represents splice mutation, red represents amplification, and gray represents no alteration. (**B**) The putative copy-number alterations of HMGA2 in mPCa patients samples treated with various chemotherapy regimens. The X-axis indicates the chemo regimen category, and the Y-axis represents the HMGA2 putative copy-number alterations. Green indicates shallow deletion, gray indicates diploid, pink represents gain, and red indicates amplification.

cBioportal serves as a free exploratory platform designed for visualizing and analyzing cancer genomic datasets. The cBioportal analysis of mPCa from SU2C/PCF Dream Team consisting of 444 samples from 429 patients reveals 3% HMGA2 alterations, which are mainly amplification and a splice mutation (Figure 5A). These patients also display 59% AR alterations (Figure 5A); 43% of the patients with HMGA2 amplification received abiraterone treatment, while 21% received enzalutamide, and 36% received MLN8237 Aurora A kinase inhibitor (alisertib), which has been utilized in clinical trials for patients with neuroendocrine features. (Figure 5B, link). Overall, 3% of HMGA2 and 59% of AR alterations are observed in mPCa, and most underwent treatment with alisertib and anti-androgen therapy.

### 3.6. HMGA2 Amplification in mPCa Is Mostly Found in Bone and Lymph Nodes and Is Associated with Tissue Exposed to Anti-Androgen Therapy

**Figure 6:**
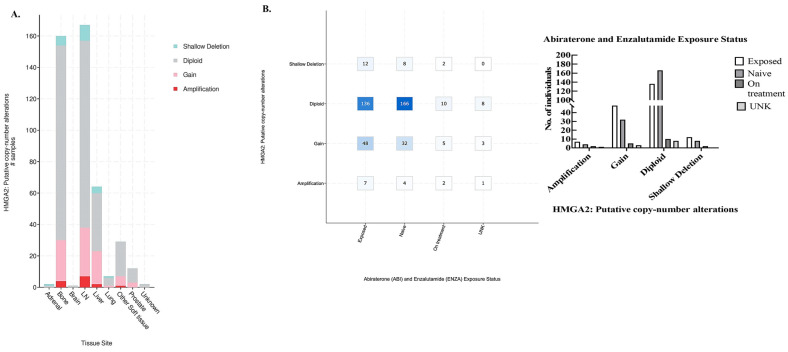
HMGA2 putative copy-number alterations. (**A**) cBioportal was utilized to analyze various HMGA2 alterations in different tissues including adrenal, bone, brain, lymph node (LN), liver, lung, prostate, etc. Green indicates shallow deletion, gray indicates diploid, pink represents gain, and red indicates amplification. (**B**) Left panel: The X-axis represents abiraterone and enzalutamide exposure status, and the Y-axis indicates the different alterations, including shallow deletion, diploid, gain, and amplification, while the number of mPCa patients is indicated in the boxes. Right panel: The X-axis represents different alterations including amplification, gain, diploid, and shallow deletions, Y-axis indicates the number of individuals exposed prior, never exposed (naïve), or currently on treatment, as well as unknown (UNK).

To further explore the clinical relevance of our findings, we turned again to cBioportal using the same PCF/SU2C mPCa dataset of 444 samples from 429 patients. Analysis of tissue sites of HMGA2 alterations revealed that most amplification and gain in HMGA2 was located in bone and lymph node tissue (Figure 6A, link). Out of the 444 samples, HMGA2 amplification is observed in 4 samples in bone metastasis and 11 samples in lymph node metastasis, which comes to a percentage of 2.5% HMGA2 amplification in bone and lymph node metastasis (Figure 6A, link). This suggests that most HMGA2 amplification is found in these sites of metastasis. Additionally, most HMGA2 alterations including amplification, gain, and shallow deletion were associated with tissue that had been exposed to abiraterone and enzalutamide therapy compared to naïve tissue (Figure 6B, link, plot type: table). Furthermore, overall survival decreases from a median of 29.50 months (95% CI 22.21–37.91) to 25.62 months (95% CI 18.43–32.13) for mPCa patients with both HMGA2 and AR alterations, although the *p* value of 0.552 is not significant (Supplementary Figure S9). Therefore, clinically, HMGA2 overexpression may be relevant in bone and lymph node metastasis and may be increased in tissue that has been treated with anti-androgens.

## 4. Discussion

Men encountering metastatic prostate cancer (mPCa) often confront significant challenges, such as developing resistance to antiandrogen therapy [26]. Our recent study reveals that the wild-type (WT) isoform of HMGA2 is associated with EMT, whereas the truncated HMGA2 isoform (TR) is implicated in increased cell proliferation and migration, which does not involve EMT in prostate cancer cells [25]. HMGA2 is reported to upregulate the Snail transcription factor, which has been shown to promote resistance to enzalutamide by increasing the expression of full-length AR and AR-V7 variants [10]. Biomarkers have been utilized to distinguish between indolent and aggressive PCa and to inform treatment options, thus reducing overdiagnosis and overtreatment [27]. In this study, we investigated the impact of HMGA2 as a biomarker in enzalutamide resistance and assessed sensitivity to alisertib treatment.

Overexpression of HMGA2 TR in LNCaP and HMGA2 WT in 22Rv1 cells led to decreased sensitivity to enzalutamide, highlighting some cell line differences. Interestingly, LNCaP HMGA2 TR cells display high levels of HMGA2 with low Snail and no difference in AR compared to LNCaP Neo; therefore, resistance to enzalutamide in these cells indicates alternative pathways from Snail–AR signaling in enzalutamide resistance. This implies that resistance to anti-androgens involves several complex mechanisms and HMGA2 signaling independent of Snail–AR signaling represents one such mechanism.

Previous studies highlight various mechanisms involved in enzalutamide resistance, which include, but are not limited to AR amplification, AR variants, AR co-regulators, glucocorticoid receptors, and EMT [28]. Additionally, the involvement of AR variants in enzalutamide resistance was emphasized in a previous study [29]; this corroborates our findings where C4-2B MDVR with high protein levels of AR variants was found to be resistant to enzalutamide treatment. When the LNCaP, C4-2B, and C4-2B MDVR cells were treated with 20 µM enzalutamide, significant growth suppression was observed in the LNCaP and C4-2B cells, but not in the C4-2B MDVR cells. Given that C4-2B MDVR cells express higher endogenous levels of HMGA2 compared to LNCaP and C4-2B cells, we speculated that HMGA2 may contribute to the resistance against enzalutamide. This speculation was confirmed when the transient knockdown of HMGA2 in C4-2B MDVR cells increased sensitivity to enzalutamide without decreasing AR expression. Since Snail expression was low in the C4-2B MDVR cells, and HMGA2 knockdown did not downregulate AR, we concluded that HMGA2-mediated resistance is independent of Snail–AR signaling in C4-2B MDVR cells.

The enzalutamide treatment led to a notable decrease in AR protein expression in the LNCaP, C4-2B, and C4-2B MDVR cell lines. This was unexpected as enzalutamide inhibits AR translocation and, consequently, prevents its transcription activity in response to R1881 [9], and in our studies, we did not perform studies with androgen. Close observation of a previous study similarly shows that a 20 µM enzalutamide dose induced a reduction in AR protein levels in both parental C4-2B and C4-2B MDVR compared to the control group treated with DMSO in the absence of androgens which supports our findings [30]. Enzalutamide partially inhibited EMT markers, such as vimentin and Snail expression.

All cell lines tested were sensitive to alisertib, and interestingly, the HMGA2 knockdown sensitized cells even further to alisertib. The overall efficacy of alisertib observed in our study corroborates with previous research demonstrating significant inhibition in proliferation and cell cycle progression in other malignancies [31,32]. The alisertib treatment also led to a decline in AR. It was previously reported that the AURKA knockdown with siRNA decreased the expression of full-length AR and its variant, while a clinical AURKA inhibitor, S1451, inhibited AR variant expression by reducing the expression of the splicing factor, ASF/SF2 [33].

The cBioportal analysis indicated patients with mPCa having 3% HMGA2 alterations, and 59% AR alterations were treated with several chemo regimens. The major alterations in HMGA2 are shallow deletion, diploid, gain, and amplification. Patients with variations in these alterations were treated either singly or with a combination of these drugs. About 43% of patients with HMGA2 amplification received abiraterone treatment, 21% were treated with enzalutamide, and 36% received alisertib treatment. Our findings in this present study suggest that alisertib (MLN 8237) might be a better candidate, as it significantly suppressed proliferation in LNCaP cells overexpressing HMGA2 WT and TR isoforms, as well as C4-2B and C4-2B MDVR expressing endogenous levels of HMGA2. Additionally, the cBioportal indicated various tissues in which alterations in HMGA2 in the patients occur. The bone and lymph nodes had higher HMGA2 alterations of gain and amplification than other tissues. In our study, we utilized LNCaP PCa cells, which are PCa cells that metastasize to the lymph node, and C4-2B cells, which are LNCaP-derived cells that metastasize to the bone. Interestingly, the cBioportal indicated increased HMGA2 alterations following treatment with ADT (abiraterone and enzalutamide). Results obtained from the database showed that patients who received abiraterone and enzalutamide treatment displayed a two-fold increment in HMGA2 amplification compared to patients who did not receive treatment. Moreover, for the gain of HMGA2 copy, there was about a 33% increase in this alteration in HMGA2 following treatment with the drugs compared to those without treatment. This suggests that ADT may elevate oncogenic markers such as HMGA2. A meta-analysis of 5427 metastatic castration–sensitive PCa (mCSPC) indicated that androgen-receptor-axis-targeted (ARAT) drugs such as enzalutamide can increase overall survival [34]; however, our study suggests that, eventually, resistance may come through AR-independent pathways.

## 5. Conclusions

Our findings indicate that HMGA2 is a biomarker associated with resistance to enzalutamide but not to alisertib. This resistance to enzalutamide is independent of Snail–AR signaling. This suggests that HMGA2 plays a specific role in the development of resistance to enzalutamide in metastatic prostate cancer (mPCa). The lack of resistance to alisertib in cells overexpressing HMGA2 highlights the potential for alisertib as an alternative treatment option. By targeting mPCa cells that are resistant to enzalutamide, alisertib could provide an effective therapeutic strategy for patients with high HMGA2 levels. The identification of HMGA2 as a resistance marker could lead to more personalized treatment approaches, improving outcomes for patients with advanced prostate cancer. Overall, our study underscores the importance of investigating alternative therapies like alisertib to overcome drug resistance and enhance the efficacy of mPCa treatment regimens.

## Data Availability

The data generated in this study are available within the article and its supplementary data files. Publicly available data analyzed in this study were obtained from cBioportal (http://cbioportal.org) accessed on 1 April 2024.

## Supplementary Materials

The following supporting information can be downloaded at https://www.mdpi.com/article/10.3390/cancers16152631/s1: Figure S1: HMGA2 increases cell proliferation and EMT markers in HMGA2-transfected 22Rv1; Figure S2: Effect of alisertib and enzalutamide treatment on HMGA2 and AR in LNCaP Neo cells; Figure S3: Effect of alisertib and enzalutamide treatment on HMGA2 and AR in LNCaP HMGA2 WT cells.; Figure S4: Effect of alisertib and enzalutamide treatment on HMGA2 and AR in LNCaP HMGA2 TR cells; Figure S5: Alisertib suppresses proliferation in C4-2B MDVR more than enzalutamide; Figure S6: Alisertib and enzalutamide treatment in LNCaP cells; Figure S7: Alisertib and enzalutamide treatment in C4-2B cells; Figure S8: Alisertib and enzalutamide treatment in C4-2B MDVR cells; Figure S9: Survival plot of prostate cancer patients with and without HMGA2 and AR alterations.
