# Supplementary material for: High Mobility Group AT-hook 2: A Biomarker Associated with Resistance to Enzalutamide in Prostate Cancer Cells
Source: Cancers (Basel). 2024 Jul 24;16(15):2631. doi: 10.3390/cancers16152631 (PMC11311100; doi:10.3390/cancers16152631)
Supplement: Supplementary file 1 [file cancers-16-02631-s001.zip › cancers-3092421-supplementary.pdf]

HMGA2: A Biomarker Associated with Resistance to Enzalutamide in Prostate Cancer Cells

Yusuf Mansur Liadi[1,2], Taaliah Campbell[3], Bor-Jang Hwang[1], Bethtrice Elliott[1] and Valerie Odero-Marah[1*]

[1]Center for Urban Health Disparities Research and Innovation, Department of Biology, Morgan State University, Baltimore MD 21251, USA

[2]Department of Biology, Umaru Musa Yar'adua University, Katsina, PMB 2218, Nigeria

[3]Center for Cancer Research and Therapeutics Development, Department of Biological Sciences, Clark Atlanta University, GA 30314, USA

*valerie.odero-marah@morgan.edu

Supplementary Material and Methods

Materials and Methods

Stable transfection of 22Rv1 cells with HMGA2 cDNA

Stable transfection of 22Rv1 cells with HMGA2 WT and TR cDNA was achieved using TurboFect transfection reagent (Catalog #: R0533) (Thermo Scientific, Waltham, MA). Following the manufacturer's instructions, cells at 90% confluency were transfected with 1.6 μg of HMGA2 WT cDNA (Catalog #: OHu25597D, GenScript), HMGA2 TR (Catalog #: OHu102359D, GenScript) or an empty vector (Neo) in 12-well dishes. Selection of stable clones was carried out with 600 μg/ml G418, and maintenance was sustained with 300 μg/ml G418. Western blot analysis was performed to validate the expression of HMGA2.

Western blot analysis

Whole cell proteins from cultured cells were extracted using a modified radioimmunoprecipitation assay (RIPA) lysis buffer containing 50 mM Tris-HCl, pH 8.0, 150 mM NaCl, 5 mM EDTA, 1% NP-40, 0.5% sodium deoxycholate, 0.1% SDS, and protease inhibitor cocktail. Protein concentrations were determined by using the biocinchoninic acid assay (BCA assay) from (Promega, Madison, WI). Electrophoresis on 10% sodium dodecyl sulfate-polyacrylamide gel was utilized to separate 20–30 μg of cell lysate, which was then trans-blotted onto nitrocellulose membrane. Subsequently, the membranes were treated with appropriate primary and secondary antibodies, and results were visualized using Immobilon Forte Western HRP substrate (Burlington, MA). For reprobing with a different antibody, the membranes were stripped using Pierce Biotechnology's Restore western blot stripping buffer (Rockford, IL).

Immunofluorescence Assay

A total of $5×10^3$ cells were plated into 8-well chamber slides (Merck Millipore, Tullagreen, Germany). The cells were fixed with methanol/ethanol (1:1 volume) and blocked with Dako Protein block (Agilent Technologies, Santa Clara, CA). The slides were incubated with primary antibody at dilutions 1:50 or 1:100 in Dako antibody diluent solution for 1 hr at room temp, washed with 1× TBS-T (Dako, Camarillo, CA), and incubated with secondary antibody in the dark for 1 hr at room temp. The secondary antibodies used include: goat anti-rabbit Oregon green 488, anti-mouse Alexa red 594 (Invitrogen, Carlsbad, CA) and rabbit anti-goat Texas red (Vector Laboratories Inc, Burlingame, CA). Slides were washed prior to counterstaining with DAPI (Invitrogen, Carlsbad, CA). Slides were mounted using Vectashield antifade mounting medium (Vector Laboratories Inc, Burlingame, CA). Fluorescence microscopy was performed using Leica DMi8 Stellaris 5 microscope and Leica Application Suite X software.

Cell Viability

Cells were seeded in a 96-well tissue culture plate at a density of 2,000 cells per well and allowed to adhere overnight. Following a 3-hour serum deprivation, the cells were treated with respective drugs (enzalutamide and alisertib). Viability was assessed 72 hours post-drug treatment using the MTS CellTiter 96® Aqueous One Solution Cell Proliferation Assay (Catalog #: G3580), following the supplier's protocol (Promega Corp).

Analysis of Publicly Available Datasets

To examine chemotherapeutic strategies for mPCa patients with HMGA2 amplification, we accessed data from SU2C/PCF, PNAS 2019, through www.cbioportal.org. Navigating to the website homepage, we

selected “Query” and then chose “Prostate Cancer (SU2C/PCF, PNAS 2019)” which included 444 samples. Within the “Select Genomics Profiles,” we opted for “Putative copy-number alterations from DNA copy” and under “Select Patient/Case Set,” we specified “All Tumors (444).” We then entered the gene set “HMGA2”, or “AR” and proceeded to “Submit Query”. Moving on to the “Plots” section, we customized the analysis further to examine overall survival.

Liadi et al., Supplemental Figure 1

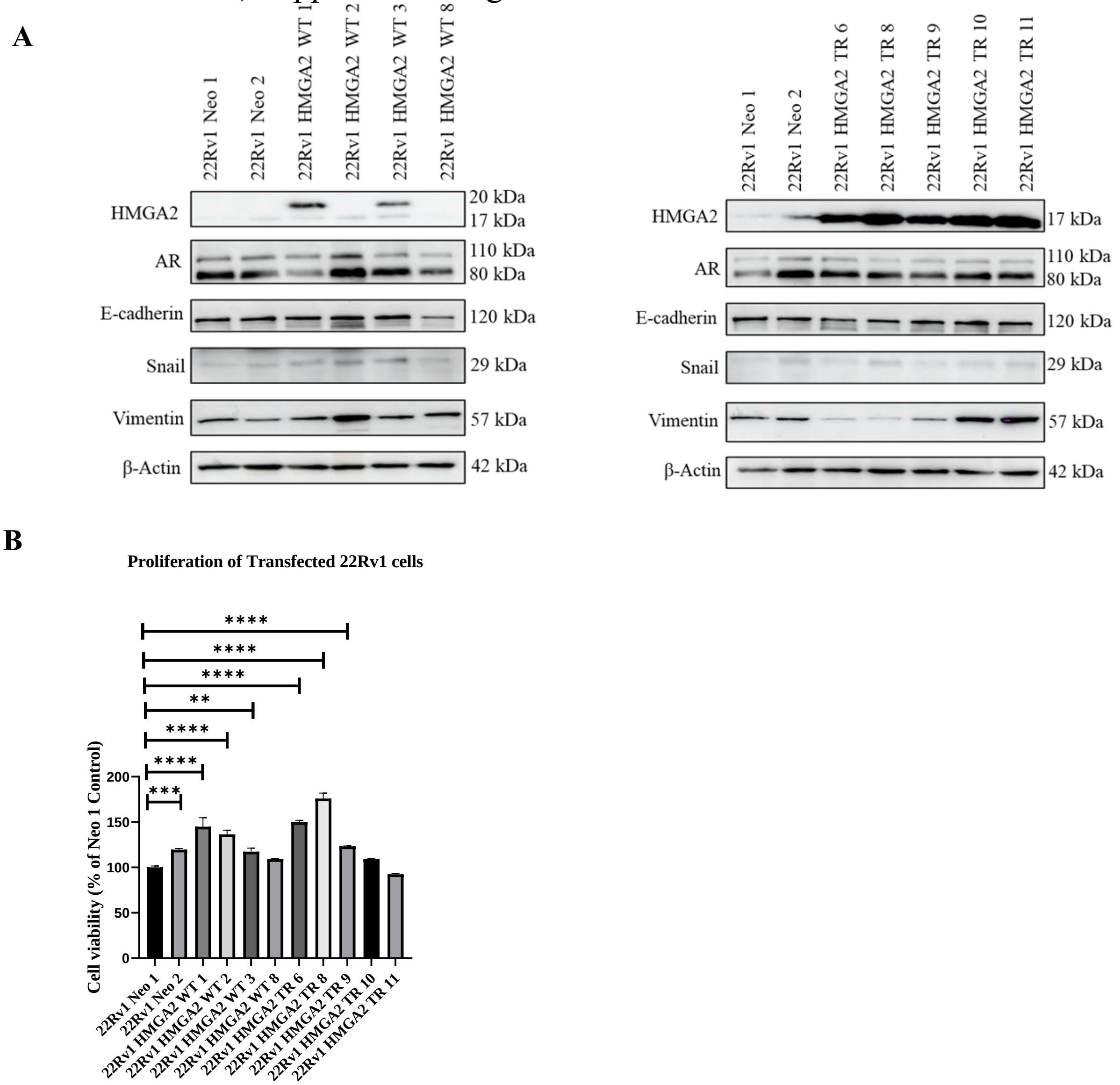

Figure S1: HMGA2 increases cell proliferation and EMT markers in HMGA2-transfected 22Rv1.
A. 22Rv1 cells were stably transfected with Neo control, HMGA2-WT cDNA or HMGA2-TR cDNA. Transfected 22Rv1 cells were analyzed for HMGA2, AR, E-cadherin, Snail, Vimentin by Western blot analysis. β-actin was utilized as the loading control. **B**. Cell viability was analyzed in transfected 22Rv1 cells. Statistical analysis was performed using GraphPad Prism, (****p< 0.0001, ***p< 0.001, **p < 0.01, * p< 0.05). Error bars show the standard deviation of the mean. The results are representative of 2 independent experiments.

Liadi et al., Supplemental Figure 2

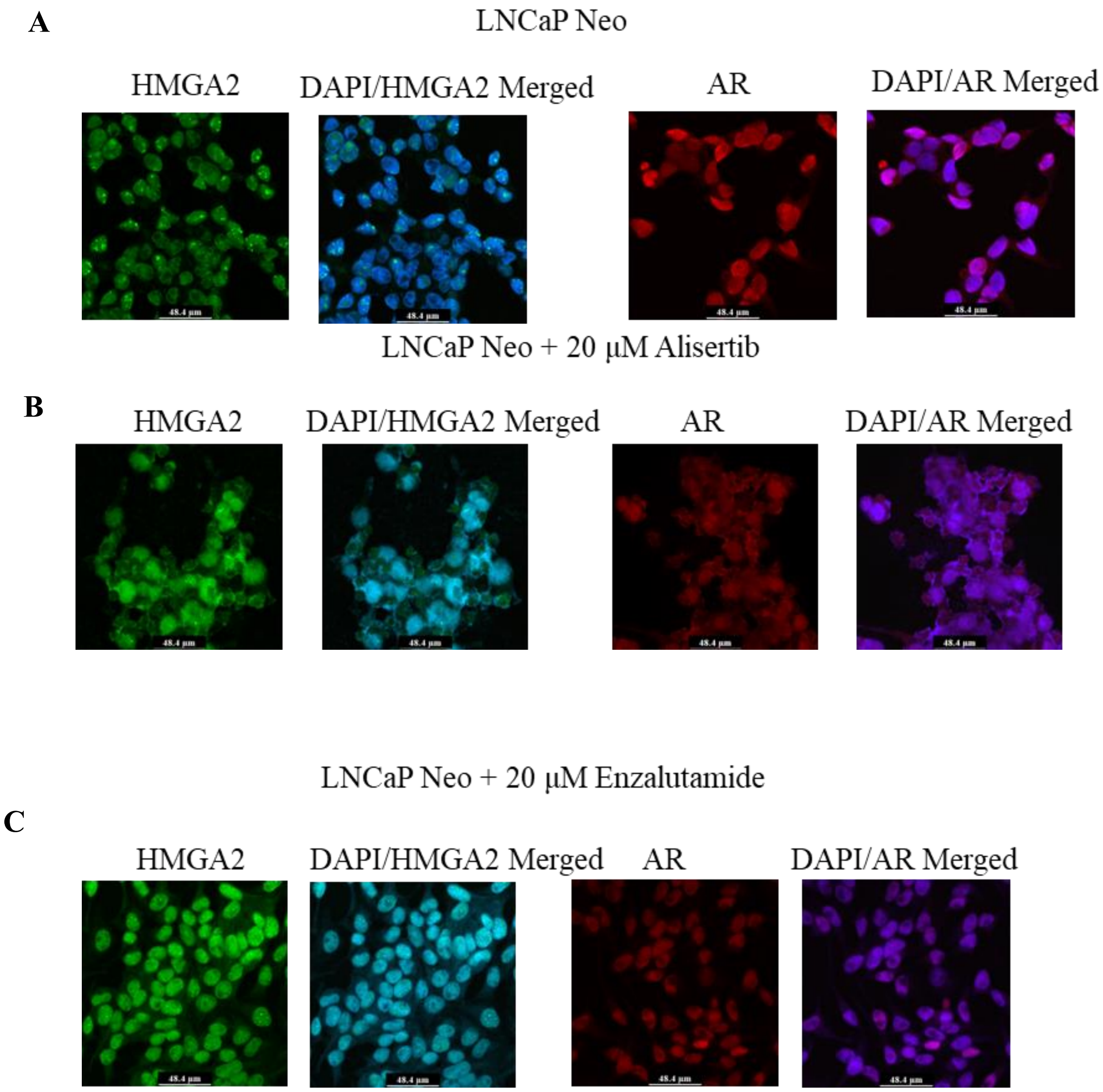

Figure S2: Effect of alisertib and enzalutamide treatment on HMGA2 and AR in LNCaP Neo cells. Representative images of immunofluorescence staining of HMGA2 and AR co-localization in **A.** Untreated LNCaP Neo cells. **B.** Alisertib-treated LNCaP Neo cells. **C.** Enzalutamide-treated LNCaP Neo cells.

Liadi et al., Supplemental Figure 3

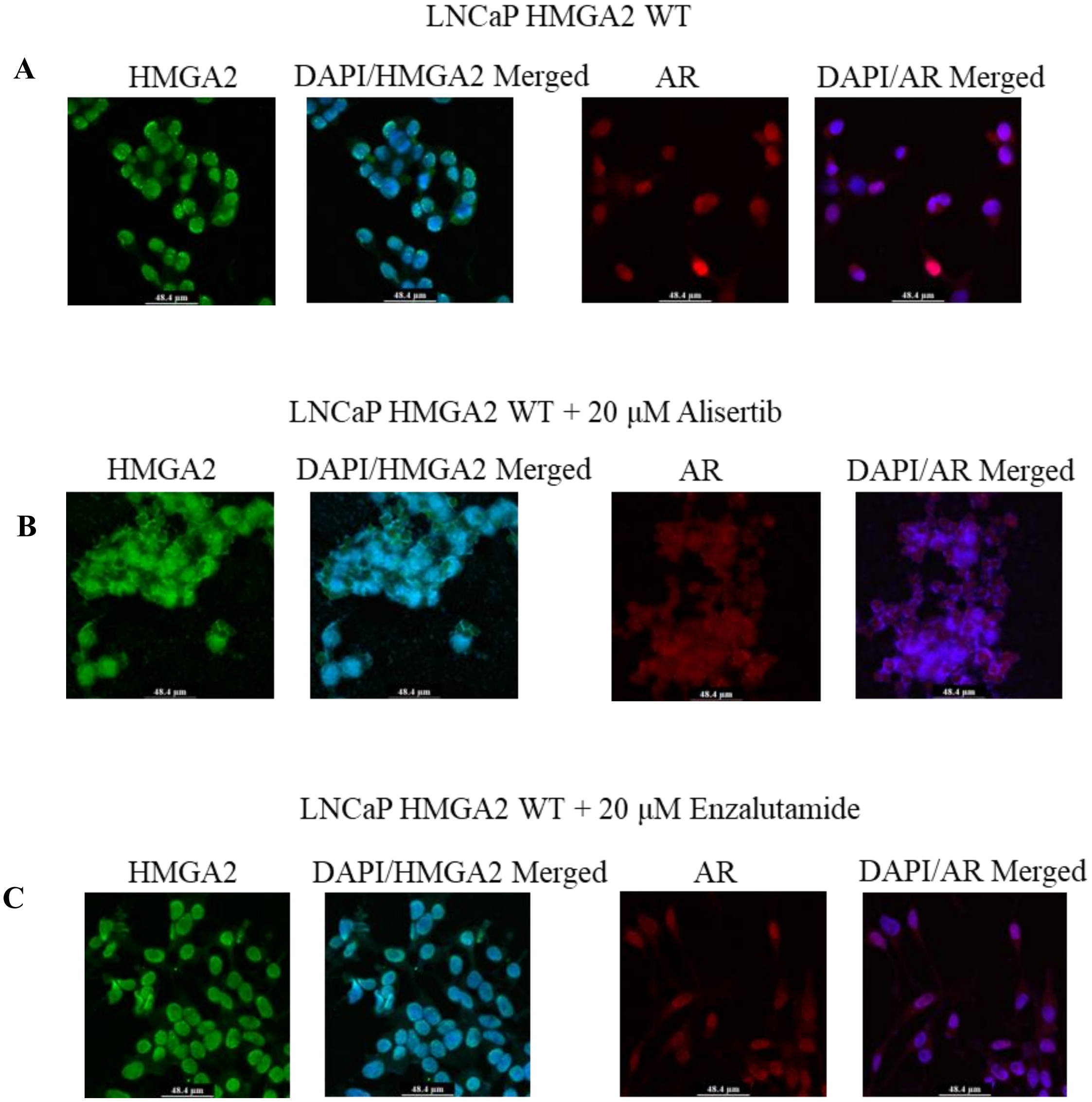

Figure S3: Effect of alisertib and enzalutamide treatment on HMGA2 and AR in LNCaP HMGA2 WT cells.
Representative images of immunofluorescence staining of HMGA2 and AR co-localization in **A.** Untreated LNCaP HMGA2 WT cells. **B.** Alisertib-treated LNCaP HMGA2 WT cells. **C.** Enzalutamide-treated LNCaP HMGA2 WT cells.

A

Liadi et al., Supplemental Figure 4

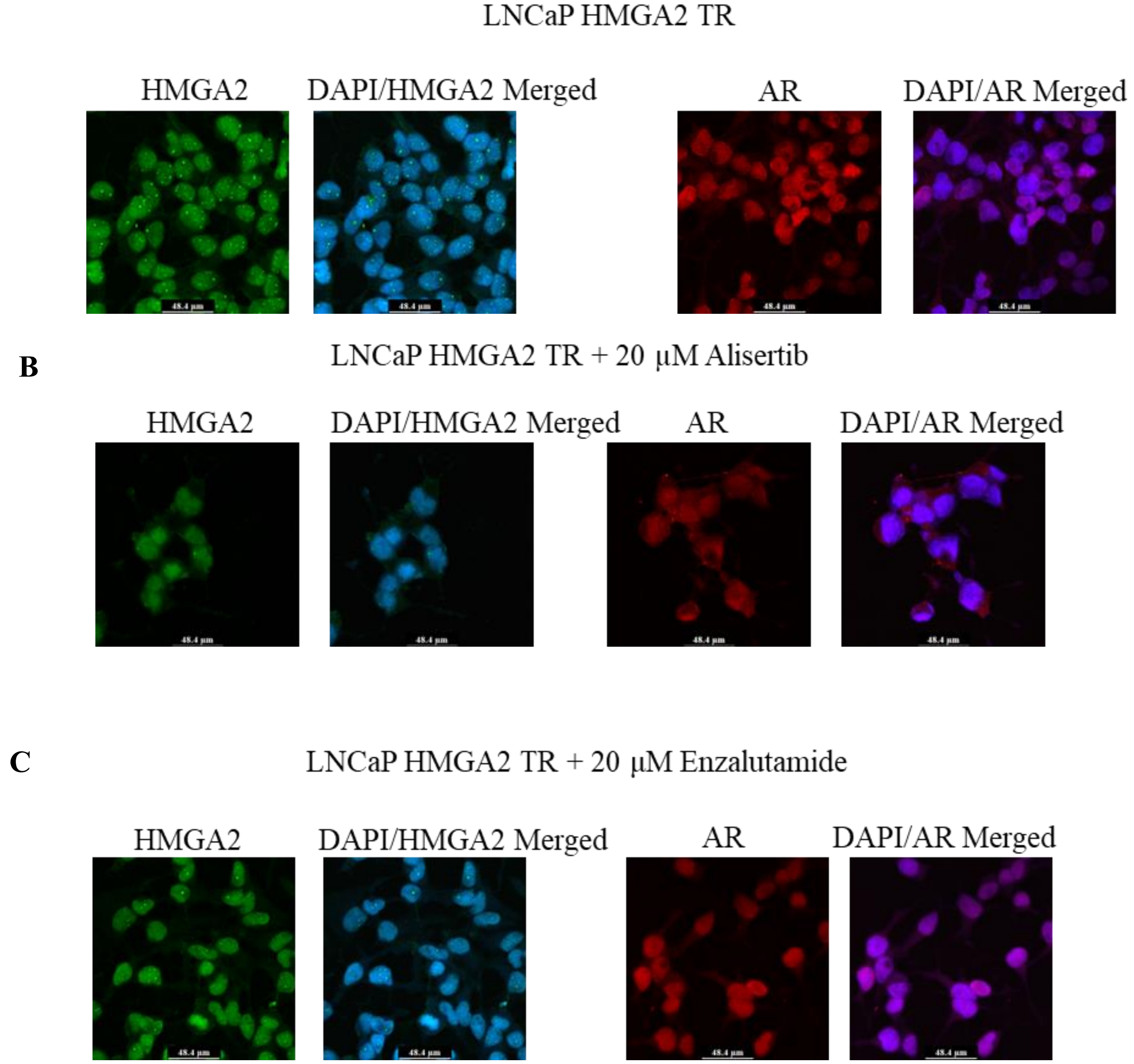

Supplemental Figure S4: Effect of alisertib and enzalutamide treatment on HMGA2 and AR in LNCaP HMGA2 TR cells. Representative images of immunofluorescence staining of HMGA2 and AR co-localization in **A.** Untreated LNCaP HMGA2 TR cells. **B.** Alisertib-treated LNCaP HMGA2 TR cells. **C.** Enzalutamide-treated LNCaP HMGA2 TR cells.

Liadi et al., Supplemental Figure 5

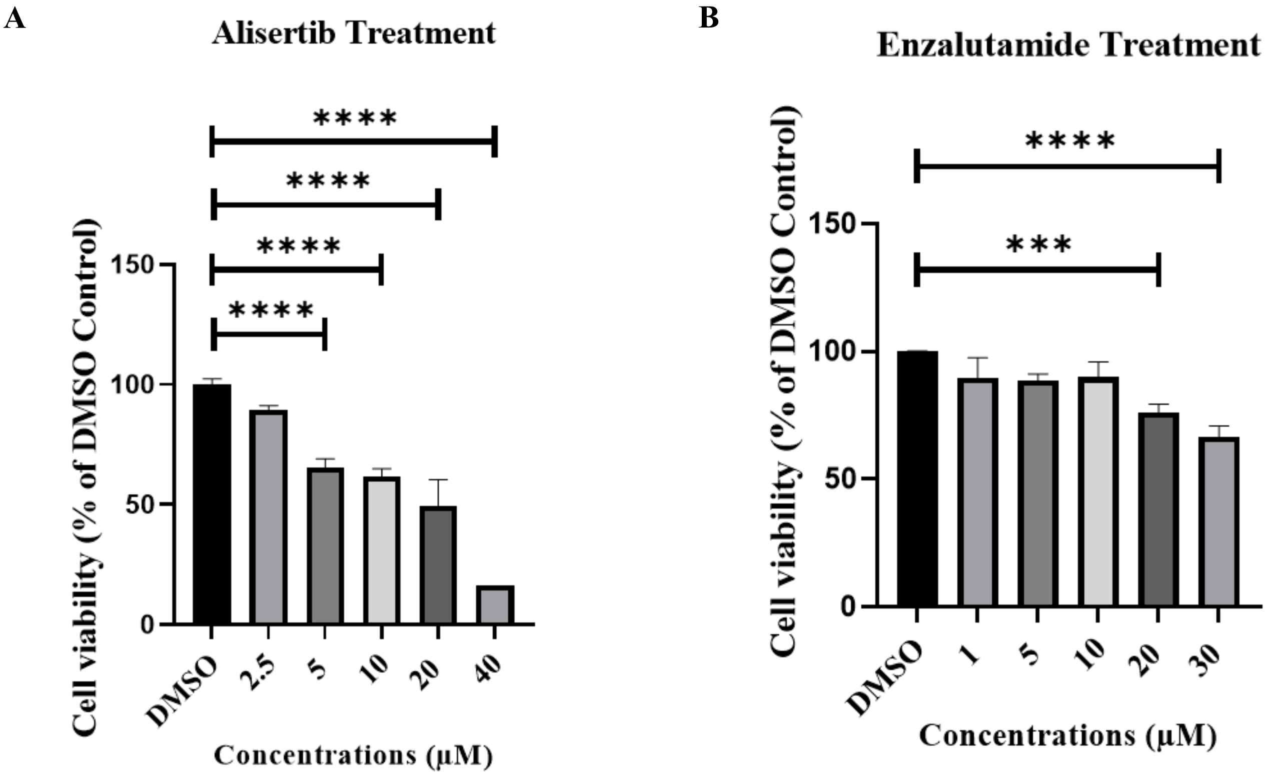

Figure S5: Alisertib suppresses proliferation in C4-2B MDVR more than enzalutamide.
C4-2B MDVR cell treated with different concentrations of **A.** alisertib or **B.** enzalutamide. Cell viability assay shows a dose-dependent decline in the cells following alisertib treatment. Statistical analysis was performed using GraphPad Prism, (****$p< 0.0001$, ***$p< 0.001$). Error bars show the standard deviation of the mean. The results are representative of 2 independent experiments.

Liadi et al., Supplemental Figure 6

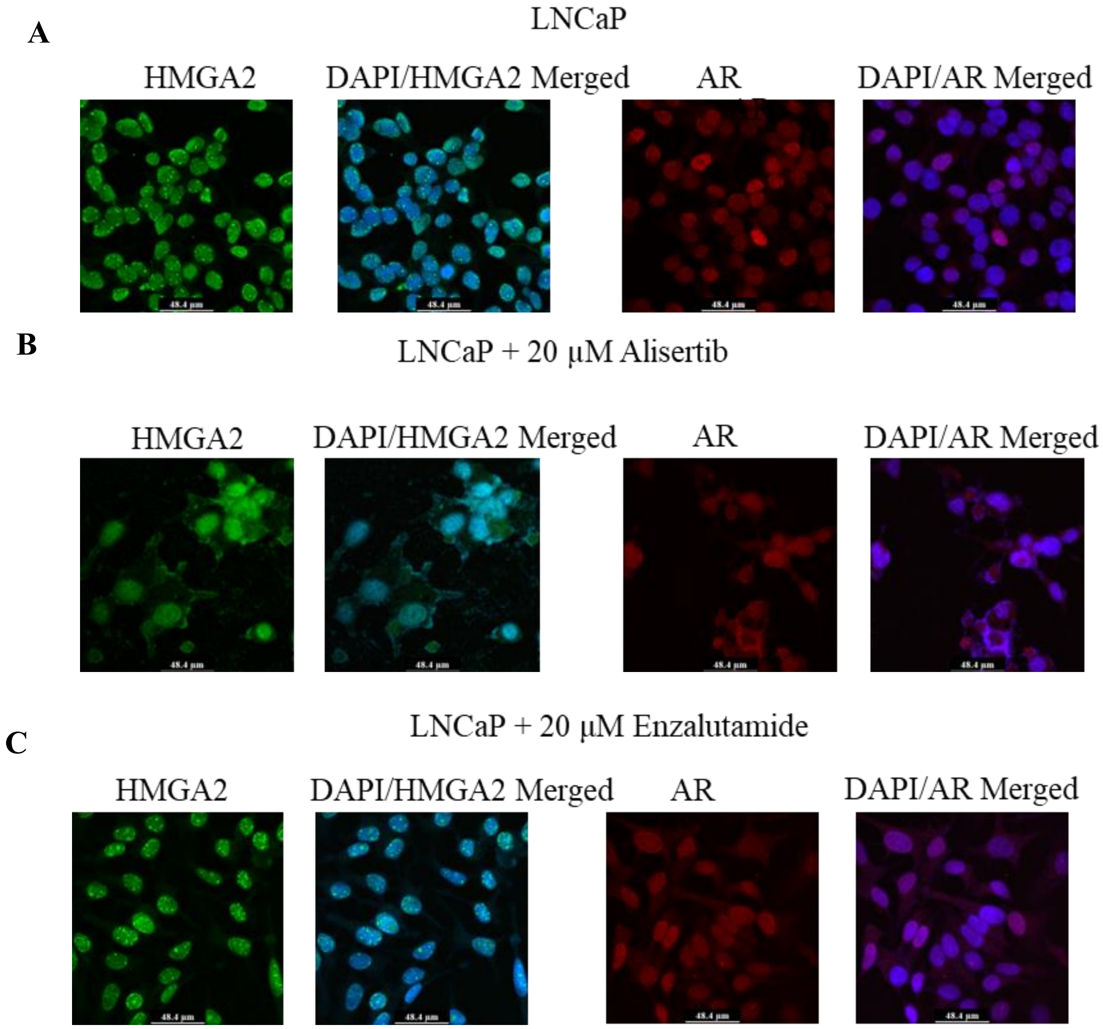

Supplemental Figure S6: Alisertib and enzalutamide treatment in LNCaP cells.
Representative images of immunofluorescence staining of HMGA2 and AR co-localization in **A.** Untreated LNCaP cells. **B.** Alisertib-treated LNCaP cells. **C.** Enzalutamide-treated LNCaP cells.

Liadi et al., Supplemental Figure 7

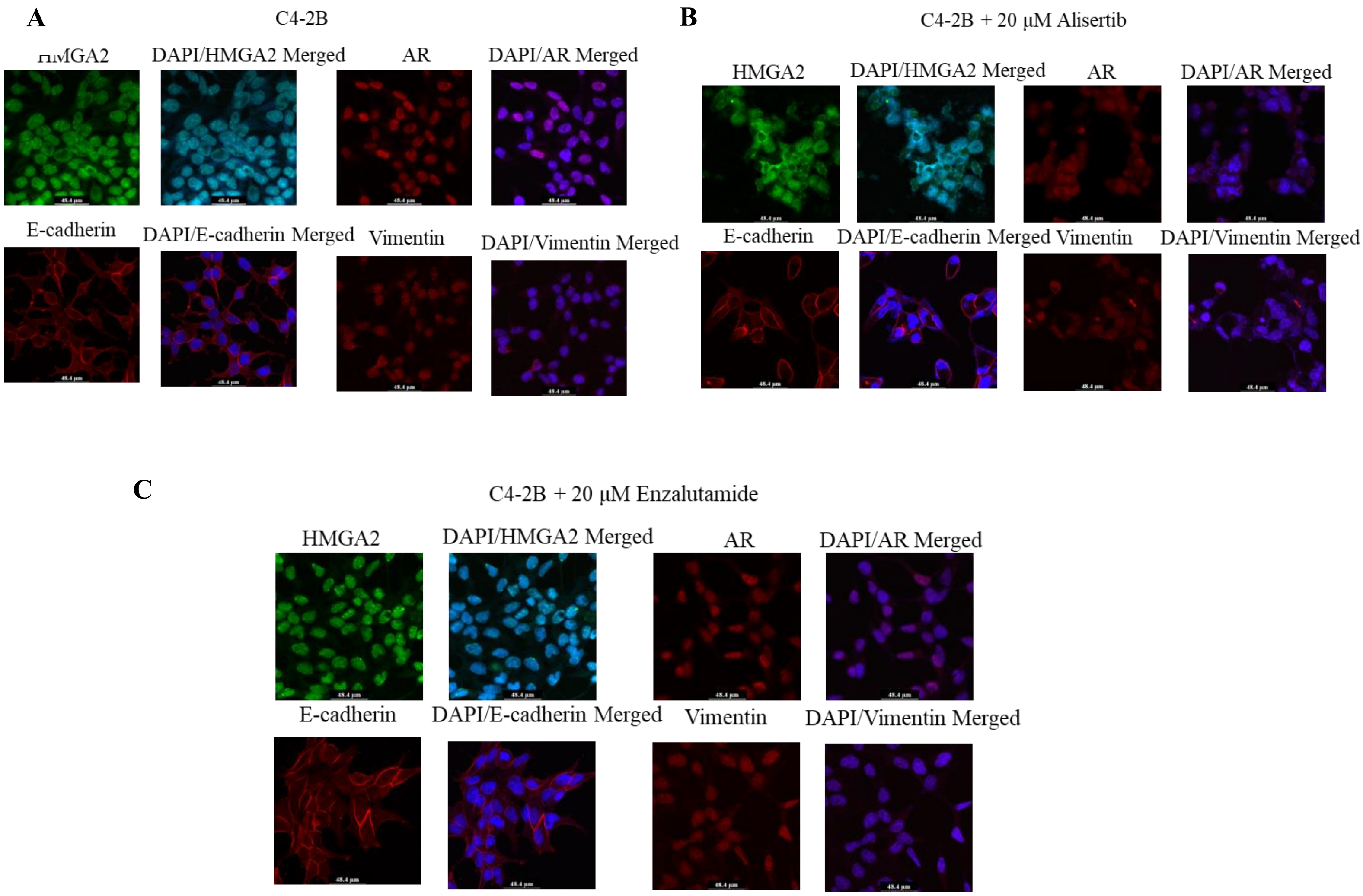

Supplemental Figure S7: Alisertib and enzalutamide treatment in C4-2B cells.
Representative images of immunofluorescence staining of HMGA2, AR, E-cadherin, and Vimentin co-localization in **A.** Untreated C4-2B cells. **B.** Alisertib-treated C4-2B cells. **C.** Enzalutamide-treated C4-2B cells.

Liadi et al., Supplemental Figure 8

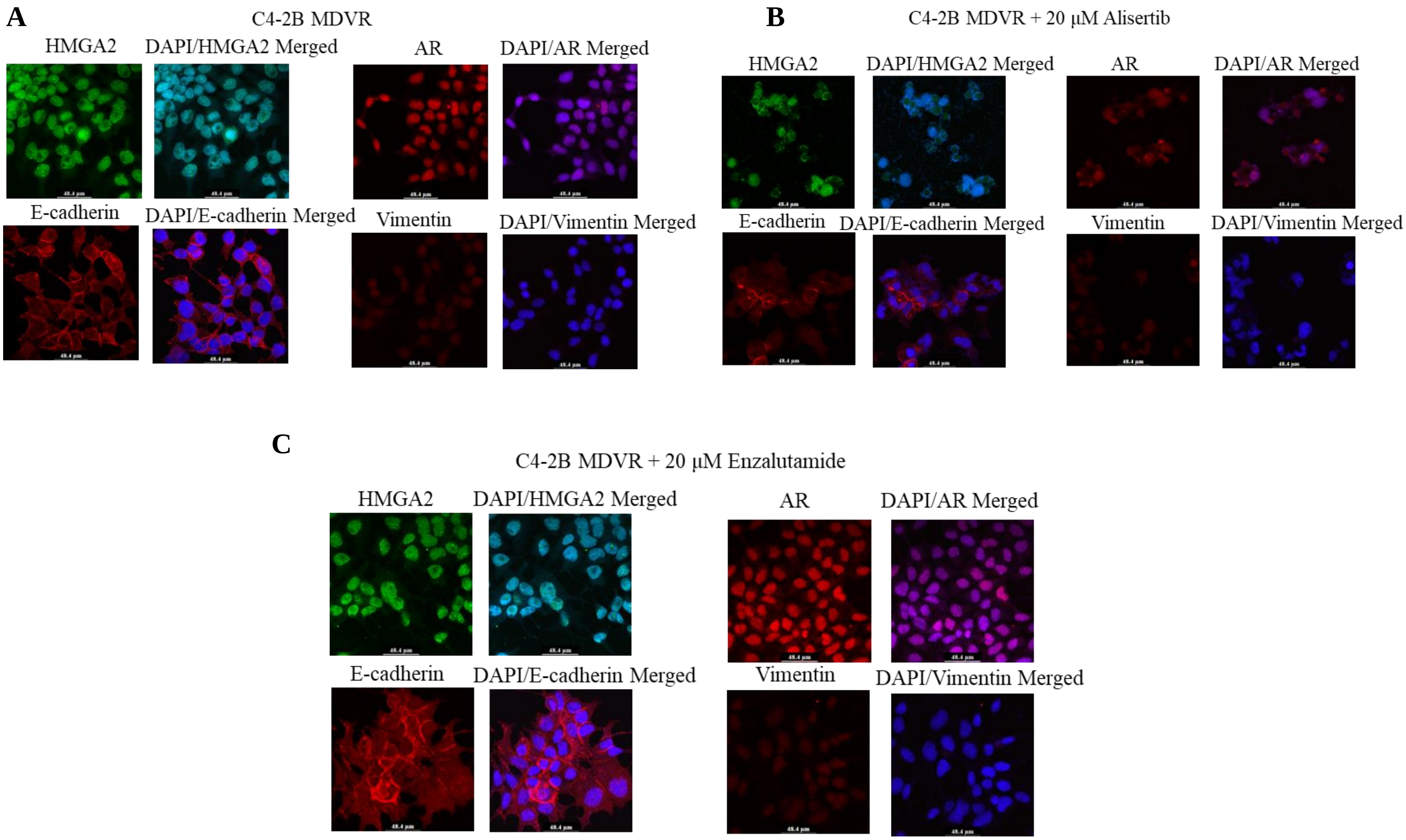

Supplemental Figure S8: Alisertib and enzalutamide treatment in C4-2B MDVR cells.
Representative images of immunofluorescence staining of HMGA2, AR, E-cadherin, and Vimentin co-localization in **A.** Untreated C4-2B MDVR cells. **B.** Alisertib-treated C4-2B MDVR cells. **C.** Enzalutamide-treated C4-2B MDVR cells.

Liadi et al., Supplemental Figure 9

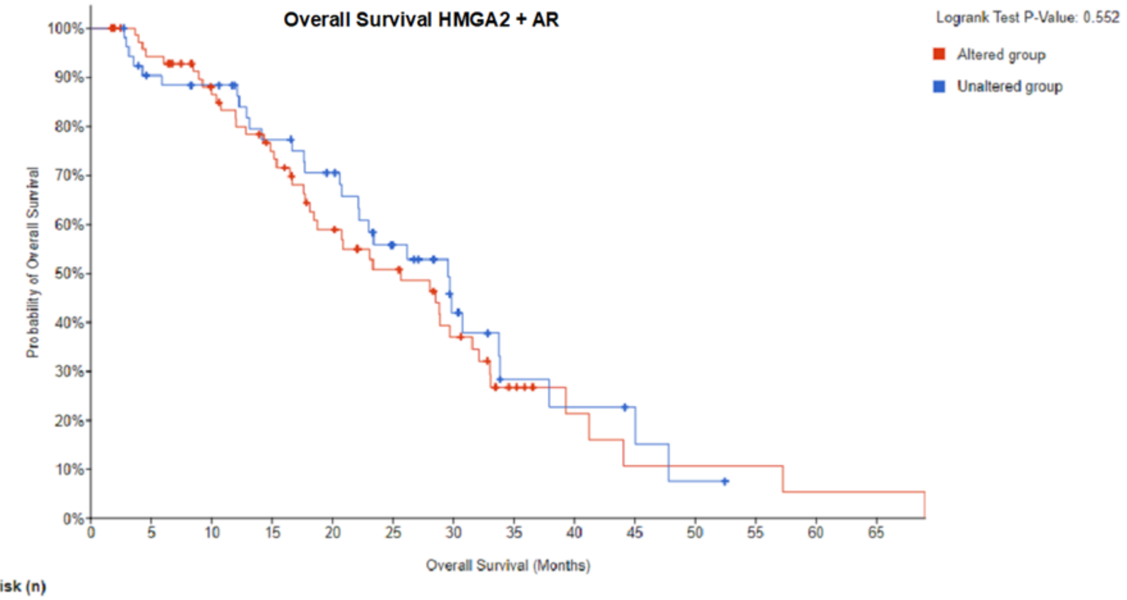

Number at risk (n)

Altered group	73	65	54	44	32	24	16	8	4	2	2	2	1	1
Unaltered group	53	45	43	35	30	20	11	5	4	3	1	0	0	0

Survival plot summary

	Number of Cases, Total	Number of Events	Median Months Overall (95% CI)
Altered group	**73**	**44**	**25.62 (18.43 - 32.13)**
Unaltered group	**53**	**30**	**29.50 (22.21 - 37.91)**

Supplemental Figure S9: Survival plot of prostate cancer patients with and without HMGA2 and AR alterations. The X-axis indicates the overall survival in months and Y-axis represents the probability of overall survival. Red represents altered group and blue represents unaltered group.
